# A Misdiagnosis of Brucellosis Leads to Prosthetic Valve Endocarditis Complicated by Cerebrovascular Accident

**DOI:** 10.1155/crdi/7361317

**Published:** 2024-11-29

**Authors:** Mahnaz Arian, Ali Tajik, Mohammad Abbasi Tashnizi, Abdul Rahman Alizada

**Affiliations:** ^1^Department of Infectious Diseases, Faculty of Medicine, Mashhad University of Medical Sciences, Mashhad, Iran; ^2^Student Research Committee, Faculty of Medicine, Mashhad University of Medical Sciences, Mashhad, Iran; ^3^Department of Cardiac Surgery, Mashhad University of Medical Sciences, Mashhad, Iran

**Keywords:** brucellosis, cardiovascular complications, case report, prosthetic valve endocarditis, stroke

## Abstract

Cardiovascular complications of Brucellosis are not common and affecting less than 2% of cases. In clinical practice, endocarditis is the most frequent cardiovascular complication and is responsible for most of the brucellosis-related mortality cases and usually diagnosed lately in the course of the disease with mostly aorta valve involvement. In this case report, we present the case of a 27-year-old woman who was admitted to the hospital with fever, sudden onset right side hemiparesis, and horizontal gaze palsy. During the stroke work up, she underwent cardiac evaluation, including echocardiography with susceptibility to septic emboli with cardiac origin, and the result indicates presence of vegetations on prosthetic aortic valve suggestive of infective endocarditis. Hopefully our patient responded well to combination of heart surgery and antibrucellosis regimen and was finally discharged with stable general condition. It is important to raise awareness of this rare but potentially serious complication of brucellosis, especially in the endemic area, and to emphasize the value of early diagnosis and treatment.

## 1. Introduction

Brucellosis is one of the most common zoonotic diseases caused by Brucella genus that primarily transmits to humans through the consumption of infected, unpasteurized livestock products or contact with infected animals, their tissues, or secretions [1]. Brucellosis occurs in all parts of the world. On a yearly basis, there are reports of 500,000 new cases, primarily concentrated in the Mediterranean, Central Asia, the Arabian Peninsula, India, and Latin America [2]. In Iran, brucellosis is widespread and remains a significant public health concern. Iran ranks fourth in the world with an average incidence of 24.43 per 100,000 people [3]. The disease can lead to various organ involvements such as osteoarticular disease, genitourinary, neurologic, cardiovascular, pulmonary, ocular, dermatologic, obstetrics, and some intra-abdominal involvements are among complications of brucellosis [4].

The cardiovascular involvement in brucellosis is reported to have an incidence rate of 1%–2% [5]. Endocarditis is the most frequent cardiovascular complication and is responsible for most of the brucellosis-related mortality cases. Due to its nonspecific clinical presentation, Brucella endocarditis (BE) often goes undiagnosed or misdiagnosed, posing a significant challenge in its management and could lead to cardiac failure which typically results in death [5]. In this case report, we present a rare occurrence of BE presenting as a stroke due to embolization.

## 2. Case Presentation

**Figure 1:**
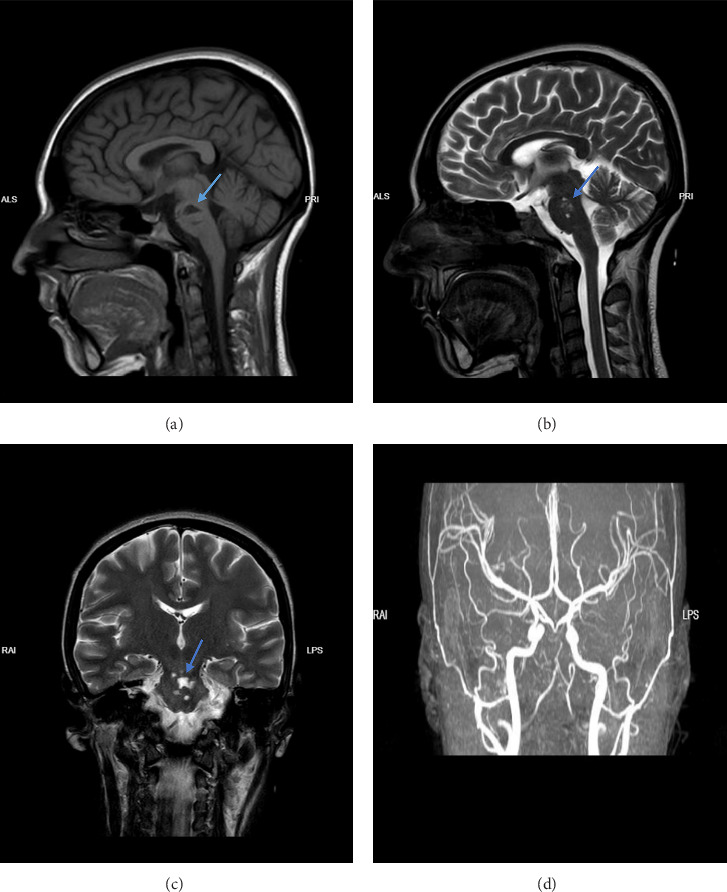
T1-weighted MRI of brain shows hyposignal areas in pons suggestive of lacunar infarction in sagittal plane. (a) T2-weighted MRI shows hypersignal areas in pons suggestive of infarction in sagittal (b) and coronal (c) planes. Brain MRA shows fetal origin of posterior cerebral arteries (PCA) bilaterally. Vertebral artery hypoplasia (VAH) was also noted (d).

A 27-year-old female patient, resident of rural areas of Mashhad, Razavi Khorasan province, Iran, working as a housewife and positive contact with livestock, with a history of open-heart surgery (biologic bileaflet aortic valve replacement and VSD repair) and a drug history of Warfarin 2.5 mg daily, was hospitalized in Mashhad, Imam Reza hospital complaining of a sudden onset right side hemiparesis and inability to move her eyes to the right. On presentation, the patient's Glasgow Coma Scale (GCS) was 15; General appearance revealed conscious, moderate nourished, intoxicated, ill female patient with pallor. Neurological exam was significant for hemiparesis and left lateral gaze palsy. Extensor plantar reflex was noted in the right foot (positive Babinski sign). Muscle force in right upper and lower extremities was 2/5 and 4/5 in left upper and lower extremities. The patient had blood pressure of 112/68 mmHg with a heart rate of 92 beats per minute. A high-pitched systolic murmur was appreciated in the aortic area on auscultation. Other systemic examination did not reveal any abnormality. Patient's lab data is on Table 1. A blood culture was obtained from patient and tested positive without exact report on the bacteria. An echocardiography noted hypermobile large filamentous mass at aortic aspect of anterior AV leaflet. On brain MRI evidence of infarction in brain stem (more exclusively, pons) was shown (Figure 1). A more precisive history taking from patient revealed that she had experienced an insidious fever which has waxed and waned for 6-7 weeks before admission. Prior to admission, the patient consulted her general physician about the episodes of fever. The physician ordered the lab tests which were within normal limits with slightly elevated alanine aminotransferase (ALT). In physical examination, a high-pitched systolic murmur was appreciated during auscultation. The physician attributed this abnormal sound to the patient's previous heart surgery and prosthetic valve. Therefore, the physician mistakenly reassured patient about her condition with a prescription for antipyretics for her fever complaint.

**Figure 2:**
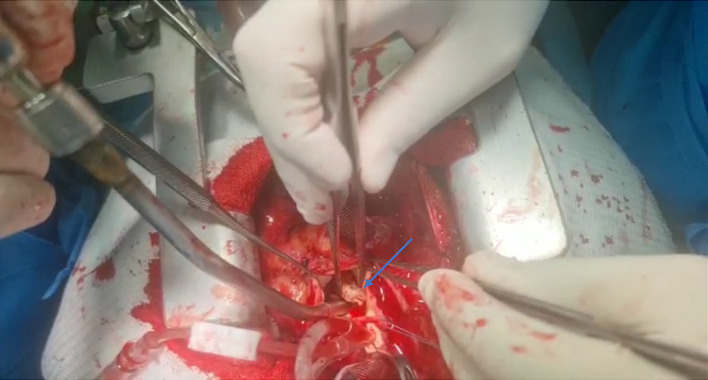
Vegetation due to Brucella endocarditis at prosthetic aortic valve in operation.

In the following, due to the patient's signs and symptoms and echocardiographic report for vegetations on prosthetic aortic valve, empiric antibiotic regimen with IV Vancomycin and Cefepime for prosthetic valve endocarditis commenced, alongside with other supportive care as needed. After 96 h of admission, patient's blood culture reported positive for Brucella spp. with consistent result in the Second obtained culture. On a second echocardiography of patient, very large multiple masses were noted on both ventricular and aortic aspect of AV mostly at aortic aspect resulting in AV stenosis; largest size of one of the masses were 29∗9∗6 mm were reported, which in comparison to the first echocardiography indicated nearly more than double enlargement in size despite starting conservative antibiotic therapy for BE. Following the blood culture and echocardiographic report, diagnosis of BE was established and prescribed antibiotic regimen changed to Doxycycline, plus Rifampin, plus Ceftriaxone. Furthermore, due to the increased size of vegetations and increased dyspnea and hemodynamic instability, alongside with positive history of open-heart surgery, cardiothoracic surgeon was consulted and an re-operative (Redo) open-heart surgery for valve replacement was planned. The patient transferred to ICU and after required evaluations moved to the operation room and valve replacement was operated. After operation, due to the bulky vegetations (see Figure 2) and history of prosthetic valve, antifungal therapy with IV Caspofungin 150 mg daily and Tab Voriconazole 400 mg bid daily in two doses and after that 200 mg bid daily was initiated for the patient due to the suspicion based on the considerable size of masses suggestive for a co-infection with fungal infections. Rifampin was also held due to the potential drug interactions with Voriconazole. Following the unfavorable pathology and lab report indicating the absence of fungal infection, the administration of antifungal treatment was ceased. After days of hospitalization, the patient reevaluated with transesophageal echocardiography which there was no evidence of vegetations or recurrency, and eventually discharged with stable general condition, normal laboratory tests, antibiotic treatment with Cotrimoxazole, Rifampin, and Doxycycline, and supportive physical therapy.

## 3. Discussion

Brucellosis (Malta fever) is a multisystemic disease that is manifested in a wide variety of ways, the most common form of presentation being fever of unknown origin, together with constitutional symptoms such as asthenia, sweating, and joint pain. Approximately 25%–35% of brucellosis cases present with focal complications, mostly the osteoarticular involvement [4]. The involvement of the cardiovascular system in brucellosis has a low rate of incidence. Endocarditis is the most prevalent cardiovascular complication and has an incidence rate of 1.3% [6]. Nevertheless, despite its low frequency, endocarditis is a very severe complication of brucellosis, since its appearance is associated with a high mortality rate in about 80% of cases [7]. The incidence of BE on prosthetic valves is 8.3% among all documented cases of BE [8]. The occurrence of acute embolic stroke as a complication of BE, as seen in our case, is extremely uncommon. In 2017, 2020, and 2024, there were three reports of a 35, 50, and a 34-year-old patient who experienced acute neurological symptoms caused by systemic emboli from BE [9–11].

Brucellosis can be difficult to diagnose due to its diverse and varied symptoms, in addition to nonspecific laboratory parameters [12]. It is crucial to perform serologic testing and culture of blood and body fluids. Among these, blood culture is considered the most reliable method. Echocardiography is essential for assessing cardiac involvement in cases of infective endocarditis, valvular destruction, aneurysm or abscess formation, or heart failure [2].

Despite its low sensitivity (15%–20%), blood culture has high specificity [13]. BE-negative blood cultures are more often due to factors like culture media, disease stage, antibiotic use, and culturing method. BE is linked to more negative blood cultures. Culture-negative endocarditis should be checked for BE. Blood culture positivity in Brucella infection is 15%–70%, but in BE it is above 80% [14].

The most effective management for BE, particularly the role of surgery, its timing, and the choice and duration of antimicrobial therapy, are unclear. Previous cases of successfully treated BE have typically involved a combination of antibiotic therapy and valve surgical intervention [15, 16]. However, cure with antimicrobial treatment alone has been reported in some cases [11, 17, 18]. The most commonly prevailing regimen is the combination of doxycycline (200 mg) and rifampicin (600–900 mg) for 10–12 weeks with aminoglycoside or third-generation cephalosporin coverage for initial 2–4 weeks [19]. The combination of antibiotic administration with valve replacement which is a very expensive option is recommended as the most effective therapy of BE [20]. Brucellosis is more prevalent in the countries with poor conditions and low income; therefore, valve replacement may not be feasible for every patient in these countries.

In this patient, in addition to the nonspecific laboratory parameters in brucellosis, the presence of a heart murmur during examination was overshadowed by the history of a prosthetic valve during the initial visit, leading to a misdiagnosis of brucellosis, which later results in complications of endocarditis and stroke. Due to the young age and multifocal signs and symptoms, one of the possible underlying etiologic causes was septic emboli from cardiac origin like endocarditis. With the history of prosthetic valve and clinical intuition, Echocardiography should be preferably performed on first admission. The current urgent or emergent indications for surgical treatment of endocarditis, regardless of antimicrobial duration, are heart failure, uncontrollable infection, and prevention of embolism [21]. The preference for homografts is largely based on their assumed high resistance to infection; nevertheless, there is no evidence supporting a substantial benefit in preventing the recurrence of infective endocarditis [22, 23]. No significant differences in survival and reoperations were observed between patients receiving an allograft and those undergoing mechanical aortic valve replacements for infective endocarditis [24]. As discussed by De Palo et al. [23], the Ross procedure may provide long-term benefits; however, in cases of uncontrolled sepsis and worsening heart failure, it is not indicated. Minimizing surgical intervention is more appropriate to enhance the likelihood of recovery and early survival. In this case, due to the evidences of heart failure and hemodynamic instability, alongside echocardiographic features of considerably large masses noted on prosthetic aortic valve and former history of stroke, surgical intervention with pre- and postoperative antibiotic coverage was preferred over antibiotic therapy alone. The decision to select a mechanical heart valve over biological one was based on its greater accessibility and other factors, such as the challenges associated with reoperation for a biological valve.

Although fungal infection was not documented for the patient during the hospital stay, antifungal therapy also prescribed for this patient on account of high clinical suspicion, as noted above, and various factors which could potentially interfere with the test result such as the specific culture media employed and the methodology.

## 4. Conclusion

Brucellosis is commonly found in developing countries; however, it is a relatively mild illness when treated correctly. One such complication like infective endocarditis related to Brucella should be suspected in patients in endemic areas for brucellosis with cardinal manifestations like fever, asthenia, and new onset cardiac murmur with a positive history of contact with livestock. Regrettably, due to nonspecific laboratory values and indistinct symptoms, it is often overlooked and can progress to severe complications, therefore requires a high index of suspicion, especially in patients presenting with stroke and fever in adolescent patients. In these cases, treatment may involve a combination of intravenous and oral antibiotics, as well as surgical intervention if presence of massive valve damage or hemodynamic instability despite medical management noted.

## Figures and Tables

**Table 1:** Patient's lab data on admission.

Test	Value	Unit	Normal range
WBC	20.4	∗1000/*μ*L	4.4–11.3
N%	86.5		
L%	8.3		
RBC	3.23	∗10^6^/*μ*L	4.5–5.1
Hb	8.7	g/dL	12.3–15.3
Hct	27.7	%	36–45
MCV	87.7	pg	26.5–32.5
MCH	26.9	fL	80–96
MCHC	31.4	g/dL	33–36
RDW-CV	16.1	%	11.5–15
PLT	177	∗1000/*μ*L	150–450
MPV	11.1	fL	8.6–12.7
BS	112	Mg/dL	
Calcium	7.7	Mg/dL	8.5–10.5
Phosphorus	7.3	Mg/dL	2.7–4.5
Magnesium	2.9	Mg/dL	1.7–2.7
Albumin	1.7	g/dL	3.5–5.5
AST	19	*μ*/L	5–40
ALT	15	*μ*/L	5–40
ALP	273	*μ*/L	< 258
LDH	709	*μ*/L	< 480
Total bilirubin	1	Mg/dL	< 1.1
Direct bilirubin	0.2	Mg/dL	< 0.4
PT			
Patient	18.6	Sec	11–13
Control	12.5	Sec	
INR	1.55		0.8–1.2
APTT			
Control	26	Sec	
Patient	41.4	Sec	25–35
ABG			
PH	7.1		Arterial blood: 7.35–7.45
PO2	47.9	mmHg	Venous blood: 7.31
PCO2	38.4	mmHg	35–45
HCO3	12.2	mEq/L	22–26
CRP	151	Mg/dL	0–8
Sodium	132	mEq/L	135–145
Potassium	5.4	mEq/L	3.5–5.3
Blood culture			
Culture 1	No growth after 72 h		
Culture 2	Brucella sp. after 96 h		
Blood culture (second time)			
Culture 1	No growth after 72 h		
Culture 2	Brucella sp. after 96 h		

Abbreviations: ALP: alkaline phosphatase, ALT: alanine aminotransferase, AST: aspartate aminotransferase, BS: blood sugar, CRP: C-reactive protein, ESR: erythrocyte sedimentation rate, Hb: hemoglobin, INR: international normalised ratio, LDH: lactate dehydrogenase, MCHC: mean corpuscular hemoglobin concentration, MCH: mean corpuscular hemoglobin, MCV: mean corpuscular volume, PLT: platelet count, PT: prothrombin time, PTT: partial thromboplastin time, RDW-CV: red cell distribution width–coefficient of variation; WBC: white blood cell count.

## Data Availability

The data used to support the findings of this study are included in the article.

## Supporting Information

As supporting information, a video depicting heart surgery is uploaded, highlighting the substantial dimensions of vegetations on the prosthetic heart valve.
